# Case report: Progressive skin rash and lymphadenopathy associated with lamotrigine−valproic acid combination in a bipolar adolescent

**DOI:** 10.3389/fphar.2023.1106423

**Published:** 2023-03-17

**Authors:** Yingxu Duan, Fangxinrui Qiu, Jingyuan Zhou, Shiyu Liu, Die Zhao, Changjian Qiu

**Affiliations:** ^1^ Mental Health Center, West China Hospital of Sichuan University, Sichuan Clinical Medical Research Center for Mental Disorders, Chengdu, Sichuan, China; ^2^ International Medical College of Chongqing Medical University, Chongqing, China

**Keywords:** lamotrigine, valproate, lymphadenopathy, bipolar disorder, case report

## Abstract

**Background:** Lamotrigine may cause severe skin reactions. There is a known interaction between lamotrigine and valproic acid with an increase in lamotrigine levels and lamotrigine toxicity risk. Few cases of severe rash and systemic reactions in bipolar patients using lamotrigine and valproate have been reported. Here, we report a rare case of severe skin rash and lymphadenopathy associated with lamotrigine−valproic acid combination.

**Case presentation:** An 18-year-old female adolescent with bipolar disorder type I was treated with lamotrigine, magnesium valproate, and perospirone for 12 days. After the last dose of lamotrigine, she abruptly developed generalized rash and swollen lymph nodes, which continued to progress over the next 3 days. This finally subsided after stopping valproate and with glucocorticoid treatment.

**Conclusion:** This case suggests that lamotrigine−valproic acid combination may cause not only rash but also lymphadenopathy. Even though the aforementioned reactions appear after the last dose of lamotrigine, it cannot be ruled out as suspicious. We recommend caution during titration of lamotrigine and valproate and early withdrawal of both when signs of hypersensitivity appear.

## 1 Introduction

Lamotrigine (LTG), a blocker of voltage-dependent sodium channels, is used as the primary therapy for focal and idiopathic generalized epilepsy (Smith, 2021). It has also been proven to be effective for treating and preventing the relapse of bipolar depression (McIntyre et al., 2020). The half-life of lamotrigine is significantly influenced when combined with other drugs. Strong genetic risk associations between HLA-B*1502 and carbamazepine-induced Stevens–Johnson syndrome (SJS) in the Han Chinese population have been identified (Chung et al., 2004). Conversely, there is a lack of evidence linking lamotrigine-induced SJS/toxic epidermal necrolysis (TEN) or drug rash with eosinophilia and systemic symptoms (DRESS) to HLA-B*1502 carriage in Han Chinese patients (Mullan et al., 2019). Valproic acid (VPA), proved to be useful in the treatment of bipolar mania or mixed states, may prolong its mean half-life by approximately two folds. There is a known interaction between lamotrigine and valproic acid with an almost two-fold increase in lamotrigine levels in patients treated with valproic acid and increase in lamotrigine toxicity risk (May et al., 1996). To date, limited reports on lamotrigine−valproate combination have shown to cause anticonvulsant hypersensitivity syndrome in only three bipolar patients and fever and severe rash in one bipolar patient (Fervenza et al., 2000; Rahman and Haider, 2005; Chang et al., 2006; Chou et al., 2014). The clinical manifestations were similarly severe or fatal, including fever, drug eruption, hepatitis, nephritis, enterocolitis, and pancytopenia. The initial dose of lamotrigine was 25 mg daily for two cases and 50 mg daily for one case. Three instances occurred during lamotrigine dosing, while one instance occurred following 1 week of lamotrigine discontinuation and start of valproate dosing, but the drug doses were not reported.

We report a case of extensive rash and lymphadenopathy in a Chinese adolescent with bipolar disorder with combined lamotrigine and valproate use.

## 2 Case description

An 18-year-old Chinese female adolescent was admitted to the mental health center ward of our hospital on 22 July 2022. She reported experiencing alternating manic and depressive symptoms over the past 5 years. She described the symptoms as excitement for 5 months manifesting as being talkative, quick to change the topic, and feeling very capable. She would then get depressed for 1 month and be crying, refusing to talk, and feeling weak. This would be followed by good mood for 6 months. The cycle persisted for the next 4 years. She was able to enter high school successfully and did not seek medical treatment. Over half a month prior to presentation, her mood deteriorated, and she attempted suicide by overdosing medication. Sometimes, she felt irritable and bit her father and herself. She was consequently diagnosed with bipolar disorder in local hospitals and treated with venlafaxine, 75 mg daily, and lithium carbonate, quetiapine, and oxazepam for 9 days. Afterward, she was asked to be transferred to another hospital, where lithium carbonate, quetiapine, and lorazepam were administered for 9 days.

During the 18 days of treatment, she had a manic episode and felt herself as intelligent as Einstein. On day 18 (14 July), medications were started as follows: lamotrigine, 25 mg twice daily; magnesium valproate, 500 mg twice daily; and perospirone, 4 mg twice daily. Her manic symptoms improved, and she was admitted to our hospital (22 July) for further consultation and treatment.

**FIGURE 1:**
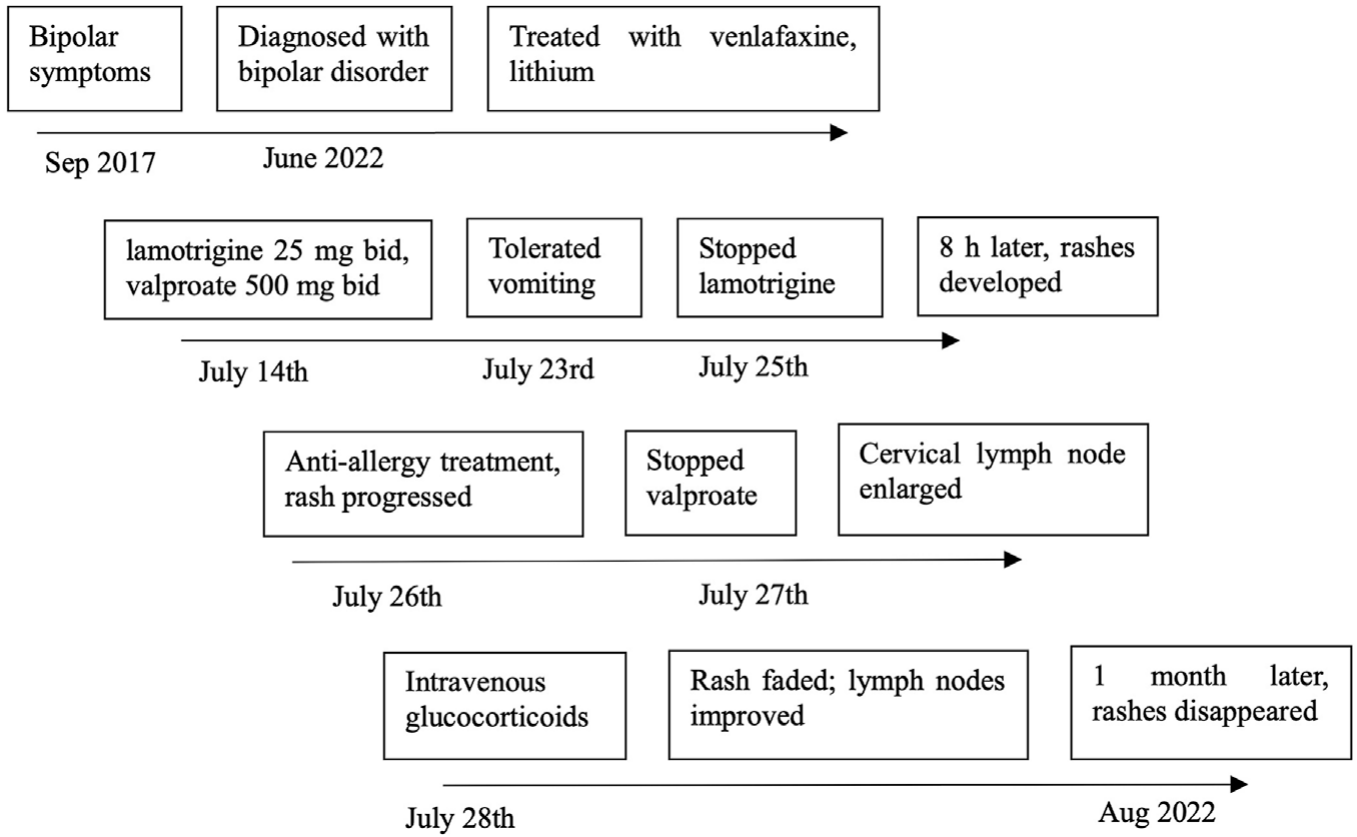
Timeline of lamotrigine and magnesium valproate dosing.

The patient’s past medical history was notable for migraine for 6 years and prior surgery for ovarian cyst torsion. She was allergic to penicillin. The patient had no family history of mental disorders. On the day of presentation (22 July), her temperature was 36.6°C, heart rate was 77 beats per minute, blood pressure was 124/70 mmHg, and respiratory rate was 20 breaths per minute (Figure 1).

## 3 Diagnostic assessment, intervention, outcomes, and follow up

After admission (after 22 July), laboratory examination showed no signs of infection and normal values for blood and urine tests, blood glucose levels, and hepatic, renal, and thyroid functions. Electroencephalography, electrocardiography, and head magnetic resonance imaging also showed no abnormalities. The patient was negative for the HLA-B*1502 allele.

Until 25 July, the patient had been taking lamotrigine, 25 mg twice daily; magnesium valproate, 500 mg twice daily; and perospirone, 4 mg twice daily for 12 days, and she experienced tolerable nausea and vomiting. We considered her medical history as alternating episodes of mild mania and depression, and that a manic episode had occurred while treating her major depressive episode with venlafaxine. We considered a diagnosis of bipolar disorder type I which was currently a manic episode in accordance with ICD-11 criteria (Organization, 2018).

**FIGURE 2:**
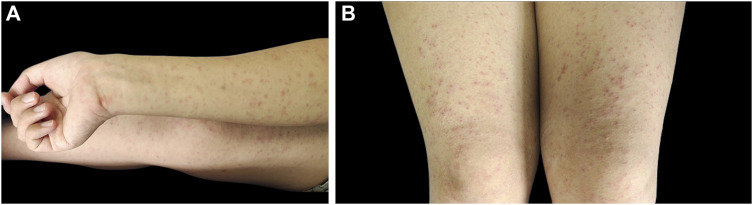
Papular rash on the right extremities **(A)** and both upper legs **(B)**.

We then discontinued lamotrigine as it was mainly indicated for treating bipolar depression. The patient suddenly developed scattered maculopapular rashes on both upper and lower extremities, 8 h following her last dose of lamotrigine. Loratadine, 10 mg daily orally, and calamine lotion for topical use were prescribed. Unfortunately, in the next 2 days, the rash gradually progressed to the trunk and neck and combined (Figure 2).

We then discontinued magnesium valproate. That evening, a lymph node on one side of the patient’s neck became visibly swollen and painful, and her body temperature briefly reached 37.2°C. Laboratory tests showed an elevation of the eosinophil percentage (10.0%). Ultrasound of superficial neck tissues showed reactive lymph node hyperplasia measuring 1 × 2 cm. The patient had prior history of mumps and the possibility of lymph node swelling due to mump recurrence was suspected, which was eventually ruled out after amylase testing resulted negative. The following day, an IV drip of methylprednisolone, 40 mg/day, was added. The rash improved, and the enlarged lymph node also decreased in size. The serum valproate concentration was 83.2 mg/L, which was equivalent to the effective therapeutic concentration. The lamotrigine concentration was not determined. Methylprednisolone was switched to oral formulation and tapered off. The medication was adjusted to quetiapine, 0.1 g twice daily. At a follow-up visit after 1 month, the patient’s rash completely resolved, and her emotions remained mostly stable. She successfully returned to school to continue her high school education.

## 4 Discussion

Our patient did not seek medical attention until her depression worsened. During treatment with venlafaxine and lithium carbonate, a manic episode occurred. This led to her drugs being switched to lamotrigine, magnesium valproate, and perospirone for mania. Venlafaxine tends to lead to a switch from depression to mania (Jacob et al., 2020). Based on DSM-5 and ICD-11 criteria, a full manic episode caused by antidepressants can be diagnosed as bipolar disorder type I (Edition, 2013; Organization, 2018). Clinical guidelines generally recommend lamotrigine as treatment for bipolar depression and maintenance but not for manic episodes (Yu and Fang, 2015; Goodwin et al., 2016; Yatham et al., 2018). The discontinuation of lamotrigine is in line with the guidelines. The patient developed rash and lymphadenopathy, 8 h after lamotrigine discontinuation, which could not be ruled out as a side effect of lamotrigine.

It is well known that lamotrigine is associated with cutaneous adverse drug reactions ranging from mild maculopapular exanthema (Shirzadi et al., 2021) to severe drug eruptions such as Stevens–Johnson syndrome, toxic epidermal necrolysis (Wang et al., 2015a), and drug rash with eosinophilia and systemic symptoms/drug-induced hypersensitivity syndrome (DIHS) (Wang et al., 2015b). The incidence of SJS, TEN, or DRESS caused by lamotrigine is estimated to be in the range of 0.01–0.1% (Mockenhaupt et al., 2005; Bommersbach et al., 2016), and that of benign rashes from lamotrigine is 10% (Wang et al., 2015c). The benign rash is usually described as a mild maculopapular rash and can disappear within a few days after stopping the suspect medication. Conversely, DRESS syndrome usually manifests as acute rash, lymphadenopathy, fever (>38°C), atypical lymphocytosis or eosinophilia, hepatitis, and multisystem involvement (Hama et al., 2022), which usually occurs 2–8 weeks after culprit drug administration (Hama et al., 2022). Of the previous four case reports of severe skin adverse reactions caused by lamotrigine−valproic acid combination, four patients developed fever and three developed extensive rashes, abnormal blood test results (pancytopenia in two patients and increase in atypical lymphocytes in one case), and abnormal liver function and were diagnosed with drug-induced hypersensitivity syndrome. One patient developed only fever and generalized rash. Other manifestations include nephritis, enteritis, cough, and headache (Fervenza et al., 2000; Rahman and Haider, 2005; Chang et al., 2006; Chou et al., 2014). Our patient had few manifestations of DRESS syndrome, such as widespread rash, enlarged lymph node, and increased eosinophil count. However, based on the diagnostic criteria of DRESS (RegiSCAR) (Kardaun et al., 2007) and DIHS (Japanese consensus group) (Shiohara et al., 2007), a diagnosis of DRESS was not established. Our patient’s rash was extensive, which progressed after the discontinuation of the drug and did not fully resolve until 1 month later, suggesting that it was not a benign rash. We suspect that these are early signs of DRESS. It may be due to the discontinuation of lamotrigine prior to the occurrence of rash. To the best of our knowledge, cases that have suspicious signs of DRESS syndrome without progressing to DRESS syndrome have not been reported previously. Our case complements this clinical information. Our patient developed rash on day 12 following lamotrigine initiation, which is consistent with that of most reports.

Lamotrigine-induced lymphadenopathy without systemic features is not common (Pomeroy et al., 2017). To our knowledge, anticonvulsant drugs causing lymphadenopathy were reported in 1940 and 1959 (Saltzstein et al., 1958). Since then, lamotrigine causing pseudolymphoma has been reported only twice (Pathak and McLachlan, 1998; Marraffa and Guharoy, 2002). In one case, lamotrigine was used continuously but lymphadenopathy remained (Pomeroy et al., 2017), suggesting that isolated lymphadenopathy may be a different process from DRESS. Fever and lymphadenopathy, however, are sometimes considered early signs of allergy. Clinicians should assess other DRESS signs, especially if the patient has recently used antibiotics, anticonvulsants, or antigout medications (Cardones, 2020). Evidently, further research is needed to explore the mechanism of lamotrigine-induced lymphadenopathy and to help distinguish benign from severe enlarged lymph nodes.

The incidence of rash following lamotrigine use may be related to the combined use of valproic acid, excessive starting dose, rapid dose increments, female gender (Wang et al., 2012), and young age (<16 years old) (Ketter et al., 2004). A possible explanation is that valproate can inhibit the glucuronidation of lamotrigine, reducing its clearance rate by approximately 50%, thereby prolonging its half-life (Kavitha et al., 2015; Koristkova et al., 2019). This effect can be maximized at a concentration of 500 mg of valproate per day (Gidal et al., 2003). A retrospective study showed that the combination of lamotrigine with valproate significantly increased the risk of rash compared to the combination with other antiepileptic drugs (May et al., 1996). Controversially, a prospective study showed that when lamotrigine was initiated at a very low dose, valproate did not cause a higher incidence of skin rashes (Faught et al., 1999). Both studies were conducted on a limited number of epilepsy patients.

Current treatment guidelines recommend a very slow up-titration when lamotrigine is prescribed alongside valproic acid to reduce the risk of skin rash (Fitton and Goa, 1995). A clinical trial of lamotrigine as a monotherapy for bipolar disorder showed that adhering to dermatology precautions with slower titration may yield a low incidence of rash (Ketter et al., 2005). In addition, data on 811 patients who took lamotrigine as monotherapy or adjunctive therapy revealed a significant correlation between lamotrigine serum concentrations and clinical toxicity (May et al., 1996). When the two drugs are combined, the recommended dose of lamotrigine in adults and children over 12 years of age should be 25 mg on alternate days for the first 2 weeks and then 25 mg daily for the next 2 weeks, with an eventual increment to 100–200 mg daily (Fitton and Goa, 1995). In our case, in combination with valproate, the starting dose of lamotrigine was 50 mg/day which is higher than the guideline recommended dose. This may be the reason for the increase in lamotrigine concentration, which further contributed to rash and other adverse reaction development.

The antimanic efficacy of antipsychotic drugs has been established (McIntyre et al., 2020), and the use of perospirone has also been described. Perospirone was metabolized by the CYP3A4 enzyme, while lamotrigine was metabolized by UDP-glucuronosyltransferase, and there is no evidence for the interaction between the two. Due to all these factors, we suspect that lamotrigine and valproate may be associated with rash development. Apparently, the most convincing way to determine the causative drug is disappearance of reactions after the drug withdrawal and reappearance of reactions when the drug is re-administered. However, based on ethical and practical considerations, we were unable to confirm this.

Of note, the rash continued to progress 3 days after stopping lamotrigine. “Prolonged clinical symptoms after discontinuation of the causative drug” is listed as one of the diagnostic criteria for DRESS. Of the previously reported four patients, three patients had progressive symptoms even after discontinuing the two drugs, manifested as fever, spreading rash, persisting diarrhea, and worsening renal function. In one patient, all symptoms resolved after the drug discontinuation. Our patient’s rash continued to progress with new lymphadenopathy after the cessation of both drugs, which supports the suspicion that it may be an early sign of DRESS syndrome. A possible explanation for this is that the half-life of lamotrigine in healthy adults is 24–35 h, which may increase to nearly 70 h when combined with valproate.

Once a rash develops, it is necessary to stop the suspected drug immediately. The use of patch testing, intradermal testing, or lymphocyte transformation testing to confirm the causative drug remains controversial (Phillips et al., 2019). Further research may consider developing a more sensitive, specific, and practical method for detecting hypersensitivity reactions to help determine the causative drug and promote early drug withdrawal and clinical improvement. Corticosteroids (intravenous methylprednisolone, 40–60 mg/d, tapered over 6–8 weeks (Shiohara and Kano, 2017)), calcineurin inhibitors, and antihistamines are the available treatment options (Cardones, 2020). In our case, we adopted these strategies and observed an early clinical improvement.

Although the pathophysiology of the drug rash due to lamotrigine is unclear, viral reactivation and human leukocyte antigen (HLA) polymorphism may be relevant. The culprit drug triggers herpes virus reactivation and in turn causes CD4 and CD8 T-cell activation (Picard et al., 2010; Miyagawa and Asada, 2021), leading to immune activation. HLA molecules on antigen-presenting cells, responsible for presenting drug antigens to their effector cells (Kano et al., 2008), may affect virus reactivation (Aihara, 2011), causing drug eruption. Patients positive for the HLA-B*1502 allele have a significantly increased risk of SJS/TEN from lamotrigine, carbamazepine, oxcarbazepine, or phenytoin (Zeng et al., 2015). In our patient, after the development of rash, we tested her for the HLA-B*1502 allele, which turned out to be negative. This does not indicate that there will be no rash or other adverse reactions (An et al., 2010). From this, we conclude that there is a need for more sensitive and specific genetic markers to deepen our understanding of the risk of severe skin reactions to lamotrigine in patients with mental disorders. Single-cell and multi-omics approaches can help us further understand, diagnose, and treat the disease (Kim et al., 2020).

In conclusion, this case suggests that lamotrigine−valproic acid combination may cause not only rash but also lymphadenopathy. Even though the aforementioned reactions appear after the last dose of lamotrigine, it cannot be ruled out as suspicious. When lamotrigine is combined with valproate, rashes and other adverse effects may progress even after the last dose of lamotrigine and can last for several days. This may relate to the effect of valproate on prolonging lamotrigine’s half-life. We recommend caution during titration when prescribing both drugs and early withdrawal of both drugs when signs of hypersensitivity appear. Future research is required to further confirm the role of the two drugs in similar skin reactions and explore early identification and judgment methods further.

## 5 Patient perspective

Originally, I was hospitalized for the treatment of bipolar disorder, but unfortunately, during the treatment, I developed a generalized rash. Luckily, this eventually got under control. I do not mind my condition being discussed by doctors all over the world since I do not want anyone to go through what I had to.

## Data Availability

The original contributions presented in the study are included in the article/Supplementary Material; further inquiries can be directed to the corresponding author.
